# Clinical SANTI classification of arthrogenic muscle inhibition has an excellent inter‐rater and intra‐rater reliability in preoperative and post‐operative anterior cruciate ligament rupture

**DOI:** 10.1002/ksa.12586

**Published:** 2025-01-16

**Authors:** Alexandre Le Guen, Emilie Bérard, Hasnae Ben‐Roummane, Kévin Lacaze, Thomas Richaud, Bertrand Sonnery‐Cottet, Etienne Cavaignac

**Affiliations:** ^1^ Department of Orthopaedic Surgery Hôpital Pierre Paul Riquet, CHU de Toulouse Toulouse France; ^2^ Department of Clinical Epidemiology and Public Health CERPOP, INSERM‐University of Toulouse III, Toulouse University Hospital (CHU) Toulouse France; ^3^ Unité de Soutien Méthodologique à la Recherche (USMR), Service d'Epidémiologie Clinique et de Santé Publique, CHU de Toulouse Toulouse France; ^4^ Sport Pro Santé Toulouse France; ^5^ Clinique Médipôle Garonne Toulouse France; ^6^ Centre Orthopédique Santy, FIFA Medical Center of Excellence, Hôpital Privé Jean Mermoz, Ramsay‐Générale de Santé Lyon France

**Keywords:** anterior cruciate ligament reconstruction, arthrogenic muscle inhibition, rehabilitation, sport surgery

## Abstract

**Purpose:**

Arthrogenic muscle inhibition (AMI) is a reflexive shutdown of the quadriceps muscles following a knee injury or surgery that presents with or without hamstring contracture. This complication can be classified according to the SANTI classification, but the reproducibility of this clinical classification has not yet been demonstrated.

**Methods:**

This single‐centre longitudinal observational study included 140 patients who were within 6 weeks of an ACL rupture. The presence of AMI was assessed separately and blindly during the preoperative consultation and at 3 weeks post‐operative by an Orthopaedic Surgeon, an Orthopaedic Resident, a Sports Medicine Physician and a Physiotherapist. AMI was also assessed a second time by the physiotherapist, 10 days after the first assessment, before and after reconstruction surgery, in order to measure intra‐rater reliability. The inter‐rater and intra‐rater reliability of the AMI classification was determined by calculating the intraclass correlation coefficient (ICC).

**Results:**

Agreement for the AMI classification between different examiners was excellent pre‐operatively (ICC = 0.99 [95% confidence interval, CI: 0.99–0.99]) and post‐operatively (ICC = 0.98 [95% CI: 0.98–0.99]). Agreement in the AMI classification, when determined repeatedly by the same assessor (physiotherapist), was excellent pre‐operatively (ICC = 0.92 [95% CI: 0.89–0.94]) and post‐operatively (ICC = 0.98 [95% CI: 0.97–0.99]).

**Conclusion:**

Excellent intra‐rater and inter‐rater reliability of the AMI classification system was found in patients with recent ACL rupture and post‐operatively.

**Level of Evidence:**

Level II, diagnostic study prospective cohort study.

**Abbreviations**
ACL: anterior cruciate ligament
AMI: arthrogenic muscle inhibition
ICC: intraclass correlation coefficient
MRI: magnetic resonance imaging
VMO: vastus medialis oblique

## INTRODUCTION

Arthrogenic muscle inhibition (AMI) can be defined as an active extension deficit at the knee due to altered contraction of the quadriceps muscles, particularly the vastus medialis oblique (VMO), originating in the central nervous system, and often associated with hamstring contracture [7, 20, 22]. Following a knee sprain, damage to the joint alters the response of sensory receptors, with profound effects on excitability in the primary motor cortex (theta waves) associated with a spinal reflex that typically produces a pattern of flexor facilitation and extensor inhibition [8, 20, 21, 22]. This pathophysiology may explain the pain‐relieving flexion contracture after a knee sprain [8, 21, 22].

If the presence of AMI is not detected preoperatively, there is a risk of post‐operative sequelae [21]. These include altered gait pattern, atrophy and chronic weakness of the quadriceps, dynamic instability, joint contracture, cyclops syndrome, persistent knee pain, proprioceptive deficits, altered motor coordination, altered movement patterns and osteoarthritis [1, 4, 9, 21]. Thus it is essential to detect, classify and treat AMI in patients who have suffered an anterior cruciate ligament (ACL) rupture [20, 21].

To be useable, a clinical classification system must be simple, reliable and reproducible [3, 16]. It must have grades that are clinically distinct and relevant to the treatment choice, prognosis and risk of complications [3, 17]. The first AMI classification was published in 2022 by Sonnery‐Cottet et al. [20, 21]. This classification is an easy tool for making a clinical diagnosis of AMI preoperatively and post‐operatively. Classifying the AMI allows a healthcare provider to determine the appropriate treatment by restoring active contraction of the VMO after ACL rupture [7, 20, 21].

To date, only inter‐observer reproducibility has been studied in preoperative ACL reconstruction, but to prove the reliability of a classification, it needs to be studied at different times, in different situations and by different users [13, 21]. To meet these criteria, we studied the classification with four healthcare professionals with different roles in the management of a patient with a cruciate ligament rupture (different users), preoperatively and post‐operatively (different situations), and with two assessments for each situation (different times).

The objective of this study was to determine the inter‐rater and intra‐rater reliability of the AMI classification used preoperatively and post‐operatively in patients having an acute ACL rupture. The hypothesis was that the inter‐rater and intra‐rater reliability of this classification is excellent.

## METHODS

The committee of our centre (*RnIPH 2023‐25*) approved this single‐centre longitudinal observational study of prospectively collected data (preoperative and post‐operative data collection). Informed consent was obtained from all patients. This study complied with the principles of the Helsinki Declaration.

Patients who suffered an acute knee injury in Toulouse between January 2023 and October 2023 and who had a consultation with a surgeon within 6 weeks of the injury were eligible for the study. Patients were included in the study if the results of a clinical examination (Lachman test, pivot shift) and magnetic resonance imaging (MRI) confirmed an ACL tear (injury to original ACL or ACL graft, as AMI has been described in both situations [21]). Patients were excluded if they were minors, if the VMO or the nerve innervating it was structurally damaged, or if they declined to participate in the study.

This study was planned and performed according to the Strengthening the Reporting of Observational Studies in Epidemiology guidelines (STROBE) [23] as well as reported according to the REporting of studies Conducted using Observational Routinely collected health Data (RECORD) Statement, an extension to the STROBE guidelines [5].

**Figure 1:**
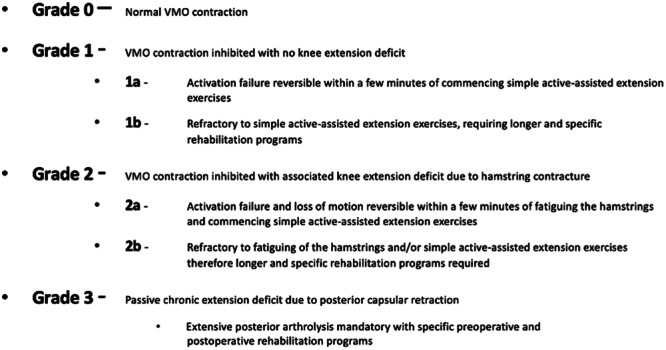
Classification of arthrogenic muscle inhibition following knee injury or surgery. VMO, vastus medialis oblique [20].

All of the included patients underwent a clinical examination during their first office visit. This consisted of a standard knee exam: AMI assessment based on the clinical characteristics of VMO inhibition, presence of an extension deficit and reversibility of these characteristics according to the SANTI group classification of AMI [20] (Figure 1), along with a Lachman test, pivot shift test and presence of intra‐articular effusion.

The AMI grade was determined independently by two orthopaedic surgeons a resident (A.L.G) and an attending (E.C.), a sports doctor (T.R.) and a physiotherapist (K.L.) successively at the patient's first visit. For the purposes of this study, the reversibility of AMI Grades 1A and 2A was defined as restoration of the patient's ability to contract their VMO normally (Grade 0) or as elimination of the extension deficit after doing simple exercises aimed at eliminating AMI during the first office visit. Release of the hamstring contracture followed by VMO awakening was done by the primary surgeon during the consultation. Its reversibility was evaluated by the different examiners by confirming recovery of full knee extension and effective VMO contraction if the patient was able to lift their heel off the exam table. Every examiner was present during the consultation and watched the patients do the exercises to awaken their quadriceps and release their hamstring. The examiners did not discuss the AMI grade with each other. This grade was recorded on a separate document for each individual examiner and was not shown to the others.

The patients were seen again by the attending surgeon 3 weeks after their surgery during a teleconsultation. This consultation was recorded so it could be viewed by the other examiners. Each examiner recorded in a separate document the post‐operative AMI grade for a given patient. The raters did not discuss the grades with each other.

To determine the intra‐rater reliability, the physiotherapist in charge of the patient's rehabilitation performed two preoperative and postoperative AMI grade assessments 10 days apart, recording the AMI grade on each occasion. This time frame was chosen because it corresponded to a patient's stable condition, and a second measurement was done blinded to the first one.

The following information was recorded preoperatively in a standardised form: sex, age, height, weight, time elapsed between injury and consultation, presence of meniscus tear on MRI, use of crutches before inclusion, use of a pillow at night under the injured knee before inclusion, use of a knee brace before inclusion, prior reconstructive surgery of a cruciate ligament. All the data were collated by the surgical resident (A.L.G.) after the consultations by gathering the data collection sheets.

The intra‐rater and inter‐rater reliability of the preoperative and post‐operative AMI classification was determined by calculating the intraclass correlation coefficient (ICC) and its 95% confidence interval (CI). The ICC values were interpreted as suggested by Landis and Koch [15]: excellent when between 0.81 and 1.00, substantial when between 0.61 and 0.80, moderate when between 0.41 and 0.60, fair when between 0.21 and 0.40 and slight when between 0.20 and 0. To show an ICC of 85% with a 95% CI of ±5% (i.e., ICC = 85% [95% CI: 80%–90%]), we calculated that at least 74 patients had to be evaluated by four examiners. The statistical analysis was done using STATA® Version 18.0 (StataCorp).

## RESULTS

One hundred forty patients were included in the study; 86 were men (61%). The patients' median age was 28.1 years. The patient‐related data are shown in Table 1.

**Table 1:** Baseline preoperative patient characteristics.

Variable	Total, *N* = 140
Sex, *n* (%)	
Men	86 (61.4)
Women	54 (38.6)
Age at inclusion	
Median [IQR]	28.1 [23.8–35.8]
Min–Max	18.1–55.8
Time elapsed between injury and preoperative consultation (days)	
Median [IQR]	31.0 [22.0–36.0]
Min–Max	6.0–42.0
ACL rupture side, *n* (%)	
Right	73 (52.1)
Left	67 (47.9)
BMI (kg/m²)	
Median [IQR]	23.9 [22.0–26.2]
Min–Max	18.2–39.5
Preoperative effusion, *n* (%)	
No	115 (82.1)
Yes	25 (17.9)
Concomitant injury, *n* (%)	
No	80 (57.1)
Yes	60 (42.9)
Did the patient use crutches preoperatively?, *n* (%)	
No	113 (80.7)
Yes	27 (19.3)
Did the patient use a pillow for support at night preoperatively?, *n* (%)	
No	107 (76.4)
Yes	33 (23.6)
Does the patient have a history of ACL rupture?, *n* (%)	
No	112 (80.0)
Yes	28 (20.0)

Abbreviations: ACL, anterior cruciate ligament; BMI, body mass index; IQR, interquartile range.

**Figure 2:**
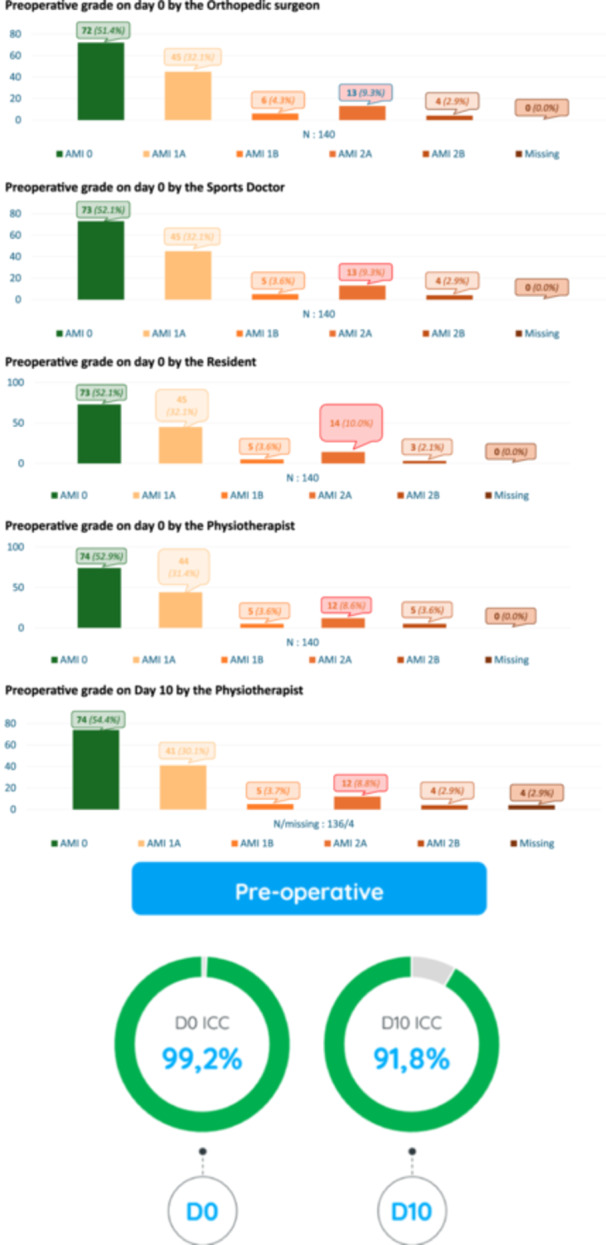
Details of the grade of AMI as reported by the observers before surgery in 140 patients and the associated intraclass correlation coefficient. AMI, arthrogenic muscle inhibition.

Preoperatively, the inter‐rater agreement for the AMI classification was excellent (ICC = 0.99, 95% CI: 0.99–0.99). Based on the attending surgeon's evaluation, 49% (*n* = 68) of included patients had AMI ≥ 1 preoperatively (Figure 2). The intra‐rater agreement for the AMI classification preoperatively was excellent when the assessments were repeated 10 days apart (ICC = 0.92, 95% CI: 0.89–0.94). The range and incidence of the various AMI grades preoperatively at Day 0 for each examiner and the repeated measurement by the physiotherapist 10 days later are shown in Figure 2.

**Figure 3:**
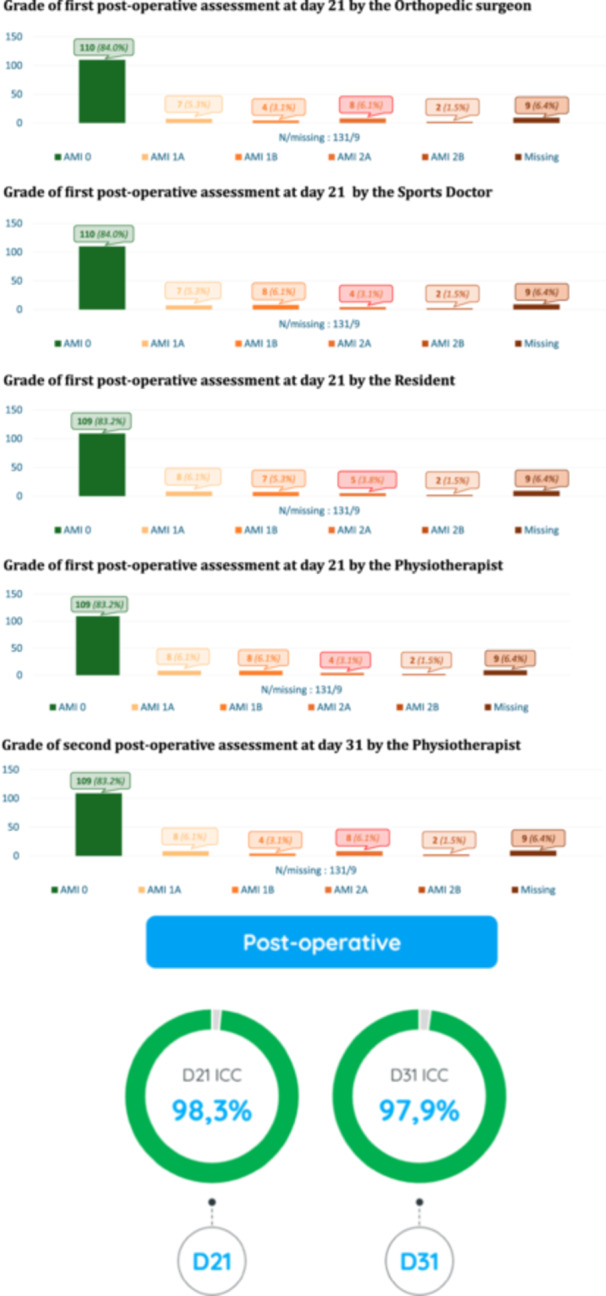
Details of the grade of AMI as reported by the observers after surgery in 131 patients and the associated intraclass correlation coefficient.

At 3 weeks post‐operative, the inter‐rater agreement for the AMI grade was excellent (ICC = 0.98, 95% CI: 0.98–0.99). The intra‐rater agreement for the post‐operative AMI classification 10 days later by the physiotherapist was excellent (ICC = 0.98, 95% CI: 0.97–0.99). The range and incidence of the various post‐operative AMI grades for each examiner and the repeated measurement by the physiotherapist 10 days later are shown in Figure 3. Based on the attending surgeon's evaluation, 16% (*n* = 21) of patients had AMI ≥ 1 post‐operatively.

The presence of AMI Grade ≥1 preoperatively was not associated with having AMI ≥ 1 post‐operatively (*p* = 0.3).

## DISCUSSION

The main finding of this study is that the clinical AMI classification developed by Sonnery‐Cottet et al. [20] has excellent intra‐rater and inter‐rater reliability both preoperatively and post‐operatively in patients with ACL rupture. This result confirms the excellent preoperative inter‐rater reliability that was previously reported [14, 21] while adding a multidisciplinary aspect and demonstrating excellent agreement when the assessment is repeated by the same examiner 10 days later.

AMI after ACL rupture is a common complication with an incidence of up to 50% reported [21]. One of the goals of this research was to educate healthcare professionals about diagnosing AMI after ACL rupture. In fact, Rush et al. reported that it was difficult for rehabilitation professionals to quantify this phenomenon due to lack of training and experience, and difficulty understanding the AMI terminology. Consequently, several rehabilitation professionals do not provide interventions to treat AMI [19]. These data highlight the need to have practical and reproducible evaluation methods for AMI management [19].

To our knowledge, this is the first study of AMI diagnosis that included not only knee surgeons but also physicians and physiotherapists who are responsible for the preoperatively and post‐operative rehabilitation of patients with ACL rupture. The excellent intra‐rater and inter‐rater reliability of the diagnosis makes it possible to validate this classification as a common language between different healthcare professionals for the diagnosis of AMI.

Once AMI is diagnosed, it can be overcome with simple in‐office exercises for easily reversible grades (1A or 2A) or during rehabilitation sessions for the more complex grades (1B or 2B) [8, 20].

As described by Sonnery‐Cottet et al, Grade 1a can be relieved by placing a cushion under the knee to allow 30° of flexion and relax the hamstrings. The patient is then asked to raise the heel, which can lead to passive contractions [20]. Then, the patient is asked to contract the muscle without lifting the heel, which can be checked by ensuring that the patella moves upwards [20]. If the patient only contracts the rectus femoris, the patella will not move upwards [20]. The thickness of the cushion is then gradually reduced, ensuring that the patient still has good contractions with each reduction [20]. Allowing the patient to palpate the proximal aspect of the patella and feel the proximal migration of the patella with each VMO contraction provides proprioceptive and visual feedback of quadriceps activation [20].

For Grade 2A, in the supine position, the examination reveals a contracture of the hamstrings on the injured side. To relieve this, the patient is asked to repeatedly contract the hamstrings against resistance, then release them completely [20]. This operation is repeated until the hamstrings are completely fatigued and the contracture disappears [20]. Once full passive knee extension has been achieved, it is important to reactivate the VMO using the exercises described above [20].

For more complex grades (1B or 2B), patients undergo specific re‐education, focusing on awakening the quadriceps using biofeedback and VMO activation [8, 18, 20].

Although we do not have sufficient follow‐up to confirm that the reduction measures described cure or definitively abolish AMI, they relieve the patient's symptoms by relaxing the hamstring muscles and restoring both knee extension and active contraction of the VMO [8].

In this study, the rate of AMI ≥ 1 was 49% preoperatively, which is similar to that in previous publications (55%) [21, 22]. This similar rate reinforces the external validity of the excellent agreement found in this single‐centre study. We found an AMI rate of 16% post‐operatively. Ours is one of the rare studies that has investigated AMI post‐operatively. Our study highlights the importance of its detection. In fact, the presence of AMI ≥ 1 preoperatively was not associated with having AMI of the same grade post‐operatively (*p* = 0.3). One possible explanation is that all the patients who had AMI preoperatively in our study participated in rehabilitation or neuromuscular reprogramming sessions if they had a Grade 1B or 2B AMI [10]. These patients used biofeedback each session until their AMI disappeared, in addition to the usual rehabilitation exercises [2, 18].

The main limitation of this classification is that it is not correlated with objective established measurements of VMO activation, for example, surface EMG [10, 11, 12, 14, 18]. One may wonder whether doing a visual evaluation only might make the diagnosis inaccurate. Nevertheless, surface EMG has inherent limitations and cannot accurately measure the activity of deep muscles [14]. The soft tissue between the electrode and target muscle can introduce interference, and the electrode could be mispositioned [14]. The measured muscle activity can also vary depending on the patient's position during the exam, and there are no EMG reference values (μV) [14, 18]. Conversely, AMI has excellent agreement when evaluated blindly by various examiners. The advantage of an exclusively clinical classification is that it is easy for any healthcare professional to use it in daily practice without devices or additional costs. Also, the examiner can detect substantial clinical improvements that could have been missed if we relied solely on EMG values.

It is important to note that none of the patients in our study had Grade 3 AMI because its diagnosis and management require a longer follow‐up than the study period allowed. Finally, these measurements were only performed in patients with ACL rupture in a single centre in order to have a homogeneous population to determine the correlation coefficient. Nevertheless, AMI can also occur in other situations such as meniscus tear or knee arthroplasty [6, 14]. As a result, this study provides a useful starting point for detecting and treating AMI, but further research is needed to show how the classification improves patient care in the long term.

## CONCLUSION

The clinical AMI classification has excellent intra‐rater and inter‐rater reliability in patients with an ACL rupture both preoperatively and post‐operatively. It is a simple and reproducible classification that can be used by the various healthcare professionals who care for patients with an ACL rupture.

## AUTHOR CONTRIBUTIONS

Bertrand Sonnery‐Cottet and Etienne Cavaignac contributed to the study proposal and design. Alexandre Le Guen led the data collection. Emilie Bérard and Hasnae Ben‐Roummane contributed to the data analysis. The first draft of the manuscript was produced by Alexandre Le Guen and revised by all authors All authors critically reviewed and edited the manuscript until it was ready for submission. Etienne Cavaignac was the senior manuscript author and is the guarantor for this study. All authors have read the final manuscript and approved it for publication. All authors contributed to the creation of this study.

## CONFLICT OF INTEREST STATEMENT

The authors declare no conflicts of interest.

## ETHICS STATEMENT

The committee of the CHU Toulouse (*RnIPH 2023‐25*) approved this single‐centre longitudinal observational study of prospectively collected data (preoperatively and post‐operative data collection). This research study complied with the principles of the Helsinki Declaration. Informed consent was obtained from all patients.

## Data Availability

Data are available upon reasonable request.
